# Perioperative factors associated with postoperative morbidity after emergency laparotomy: a retrospective analysis in a university teaching hospital

**DOI:** 10.1038/s41598-020-73982-5

**Published:** 2020-10-12

**Authors:** M. Ahmed, E. Garry, A. Moynihan, W. Rehman, J. Griffin, D. J. Buggy

**Affiliations:** 1 Department of Anaesthesiology and Perioperative Medicine, Mater Misericordiae University Hospital, Dublin, Ireland; 2 Clinical Surgery, Mater Misericordiae University Hospital, Dublin, Ireland

**Keywords:** Predictive markers, Prognostic markers, Risk factors, Gastrointestinal diseases

## Abstract

Emergency Laparotomy (EL) is associated with significant morbidity and mortality. Variation in practice and patient outcomes for patients undergoing emergency laparotomy has been identified through the UK National Emergency Laparotomy Audit (NELA), with 30-day mortality ranging from 11 to 15%. A correlation between preoperative haemodynamic parameters and increased postoperative mortality has been demonstrated by both NELA and other observational studies. The association between intraoperative haemodynamic parameters and overall postoperative morbidity has not been evaluated in EL patients. The aims of our study were to investigate the association between perioperative haemodynamic and logistic parameters and postoperative morbidity in a tertiary referral university hospital; and to compare our outcomes to that of the NELA data. A retrospective analysis correlating a range of perioperative parameters with Comprehensive Complication Index (CCI) among 86 patients who underwent EL during 2018 was conducted. Mean age was 64 years (SD 16). Median CCI was 27 [9–45], and 30-day mortality was 11.7%. Several intraoperative parameters correlated with CCI on univariate analysis. On multivariate analysis, ASA status (*P* = 0.005) and unplanned escalation to postoperative intensive care (*P* = 0.03) were independently associated with CCI. Our study shows a correlation between ASA status and unplanned escalation to ITU with increased postoperative morbidity in patients undergoing emergency laparotomy. We did not demonstrate an independent correlation between intraoperative parameters and postoperative morbidity. These findings warrant confirmation in a larger scale observational study. Outcomes in our institution are comparable to those seen in the NELA.

## Introduction

Emergency Laparotomy is a complex and often time-critical surgical procedure associated with significant morbidity and mortality. Care of patients who undergo emergency laparotomy often involves input from multiple specialties including Emergency Medicine, Surgery, Anaesthesiology, Radiology, Critical Care Medicine and Medicine for the Elderly. The UK National Emergency Laparotomy Audit (NELA) has identified a wide variation in practice and patient outcomes following this surgical operation, with an overall 30-day mortality ranging from 11 to 15%^1^. Analysis of this large database has enabled the development of a novel risk adjustment model for calculating an individual patient’s 30-day mortality^2^. It has also helped identify a number of organisational factors and hospital characteristics that are associated with improved survival in these patients^3^. Furthermore, NELA has shown a reduction in mean 30-day mortality from 11.8 to 9.6%, since the project commenced in 2013^4^. A recent analysis of an Irish university teaching hospital reported an overall 30-day mortality of 7% after emergency laparotomy, but this increased to 20% in patients aged > 80 years^5^.

Further observational studies have shown correlations between preoperative haemodynamic parameters (pulse pressure < 53 and > 62 mmHg) and increased postoperative morbidity and Myocardial Injury after Non-Cardiac Surgery (MINS)^6,7^. A large systematic review of 42 studies identified an association between intraoperative mean arterial blood pressures (MAP) < 80 mmHg for > 10 min, or < 70 mm Hg for a short duration was associated with adverse postoperative outcomes including increased mortality, MINS, Acute Kidney Injury (AKI), Myocardial Infarction (MI), and Length Of Stay (LOS)^8^. However, no study has evaluated these and other intraoperative haemodynamic parameters in terms of their association with overall postoperative morbidity in emergency laparotomy patients.

The Mater Misericordiae University Hospital (MMUH) undertakes approximately 80–90 emergency laparotomies per annum. Our objectives were firstly, to investigate the association between routine intraoperative haemodynamic parameters, measured electronically during anaesthesia, and postoperative morbidity outcomes. Secondly, to compare our service and processes to the UK NELA data as an international benchmark in terms of organisational factors and overall morbidity and mortality.

## Methods

This was a retrospective analysis of all emergency laparotomy patients presenting between January 1 and December 31 2018 at Mater University Hospital Dublin, a tertiary referral university teaching hospital. Mater Misericordiae University Hospital Research Ethics Review Board approval was obtained with a waiver of consent as this was a retrospective study. It was conducted in accordance with the principles of the declaration of Helsinki and institutional guidelines.

Eligible cases were identified by searching a prospectively maintained electronic theatre database. All patients registered as undergoing exploratory laparotomy in 2018 were included for further evaluation. Patients who underwent non-emergency, non-GI surgery were excluded. Additionally, patients undergoing laparoscopic surgery were excluded due to concern that this would result in confounding when assessing the factors affecting postoperative morbidity and mortality. Laparoscopic patients are anticipated to have lower morbidity and mortality. Cases of penetrating trauma were also excluded due to the heterogenous nature of these operations. The remaining cases went forward for further analysis.

Researchers collected a detailed and standardised dataset from patients’ medical records, from their admission through the perioperative period and their in-hospital course, including postoperative morbidity (defined by the Comprehensive Complications Index, CCI, derived from the Clavien–Dindo grading system). A higher score indicates a greater number and impact of postoperative complications. For example, a patient who developed a postoperative pneumonia which responded to oral antibiotics would receive a lower score than a patient whose postoperative pneumonia required ventilatory support in an intensive care unit.

Where patients had left hospital within 30 days after surgery, their outcome was determined from electronic records and general practitioner contact. Intraoperative haemodynamic parameters and other details were obtained by scrutinising patients’ electronic anaesthetic records recorded on the hospital Centricity system.

In addition, we assessed a number of organisational factors. These included, use of Computer Tomography (CT) imaging, consultant reporting of imaging prior to surgery, time to theatre and availability of a critical care bed postoperatively. The intraoperative record was reviewed with particular attention to haemodynamic parameters, fluid intake, vasoactive medication usage and blood product requirements. We investigated if a correlation existed between intraoperative haemodynamic parameters and postoperative morbidity. Postoperative morbidity was measured using the CCI.

Postoperative complications were identified by review of patients’ medical records by researchers, using our hospital’s electronic patient record database. Patient progression was monitored for the entirety of their hospital stay or for 30 days post operatively (whichever was longer). We used the Clavien–Dindo Classification system from which CCI is derived^9^. We defined a postoperative complication as any deviation from the ideal postoperative course, not inherent in the procedure itself and does not constitute a failure to cure. CCI scores were calculated using the online CCI Calculator at https://www.assessurgery.com/about_cci-calculator/.

If patients were cared for in an intensive care or high dependency unit postoperatively, we reviewed the critical care electronic record to assess major morbidity. We measured the duration of vasopressor use, requirement for renal replacement therapy, rate of re-laparotomy and the total length of stay in critical care. For ward based patients, discharge summaries were reviewed for postoperative complications.

The primary clinical outcome was CCI score.

Secondary clinical outcomes included 30-day mortality, LOS and requirement for renal replacement therapy, in addition to logistical parameters.

### Statistical analysis

Data was collected on a password protected shared Excel spreadsheet (Microsoft) and imported into GraphPad Prism v8 for statistical analysis. Baseline patient characteristics, intraoperative haemodynamic parameters and surgical and process details were presented as descriptive statistics and evaluated for distribution. Normally distributed data was expressed as mean (SD), and non-normally distributed data was expressed as median (inter-quartile range, IQR). Incidences of categorical data were expressed as number and percentage.

Because the primary outcome measure was the extent of morbidity, CCI was the dependent variable. Correlation between unadjusted measured variables and CCI was undertaken by univariate analysis initially, using Pearson’s correlation co-efficient for normally distributed variables and Spearman’s rank correlation co-efficient for non-normally distributed data.

A multivariable linear regression model was constructed to determine the interactive association between variables with highest indices of correlation to CCI. Based on our projected sample size of 90–100 patients for open laparotomy per calendar year, and mindful of the principle of ensuring not less than 10 subject observations per variable^20^, we included only variables with the highest extent of correlation with postoperative morbidity on univariate analysis (*P* < 0.01) which were also on the following list of a prior determined auditable parameters based on available evidence and biological plausibility:

Age, ASA status, urgency of surgery, lowest intraoperative systolic arterial blood pressure (SABP) and mean arterial blood pressure (MAP), intraoperative blood products volume, urgency of surgery, preoperative documented coronary or respiratory disease, intraoperative use of vasopressors or inotropes, siting a large bore central venous cannula, need for renal replacement therapy postoperatively, and escalation of postoperative care to intensive treatment unit (ITU) or high dependency unit (HDU) bed. ASA status was determined on the basis of clinical judgement considering the patient’s clinical condition on presentation regardless of previous co-morbidities, in keeping with the 2015 updated definitions.

All the risk factors that met these criteria were considered for the multivariate model. Backwards selection was used to restrict this model to include the main effects of *P* < 0.05 or less, data was displayed as estimate (correlation co-efficient) and 95% confidence intervals (CI).

Separately, a multivariate logistic regression model using backward selection was constructed from these candidate variables with 30 day mortality as the dependent variable. The model was limited to the variables listed above with the greatest correlation on univariate analysis. Data is displayed as Odds Ratio (OR) with 95% CI.

## Results

**Figure 1:**
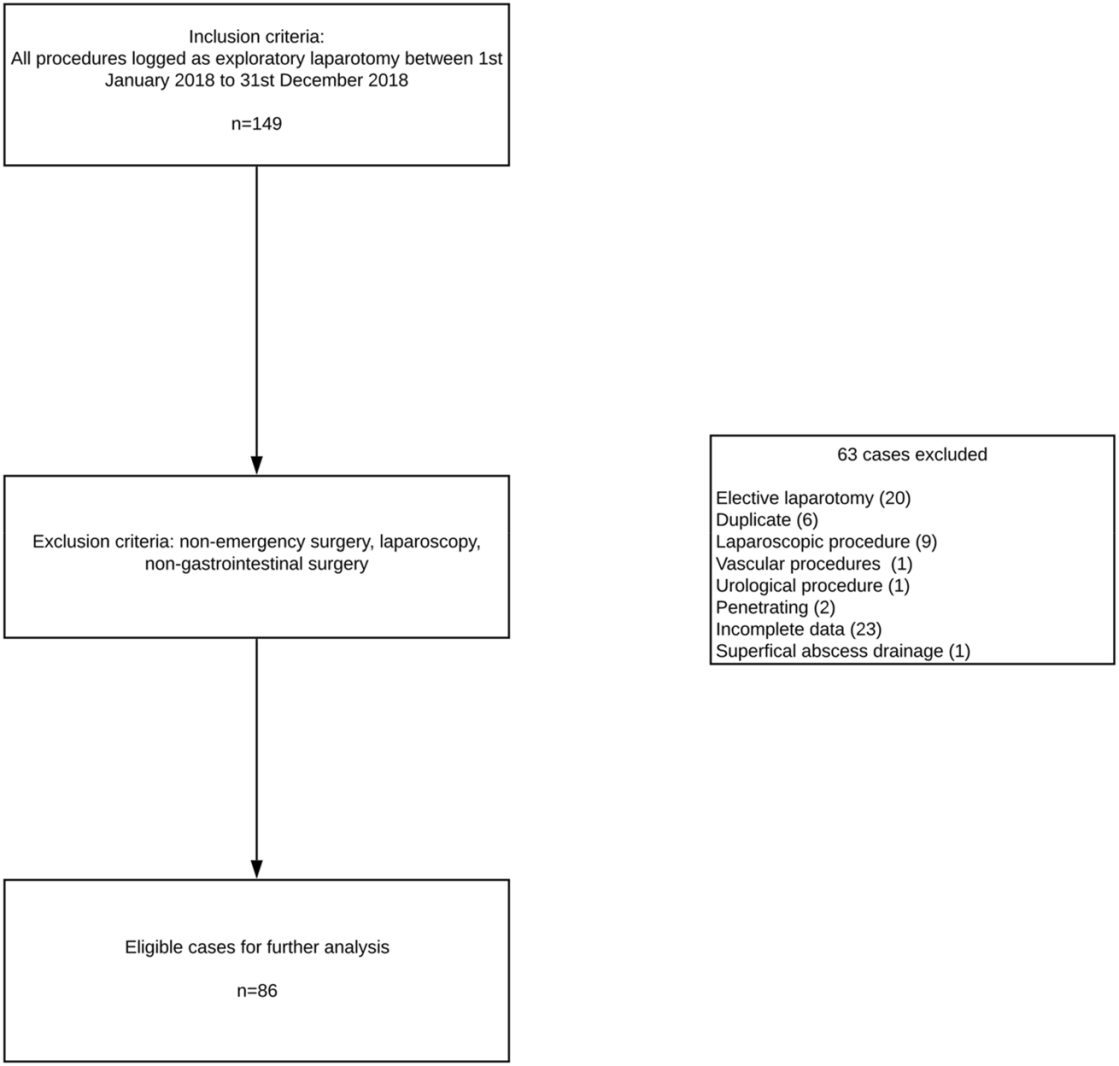
Flow diagram demonstrating inclusion and exclusion criteria.

Initially, 149 patients were identified has having undergone exploratory laparotomy in 2018. 84 patients were excluded as having had non-emergency surgery, laparoscopy or a non-gastrointestinal procedure as outlined in the study flow diagram (Fig. 1). The final dataset was n = 86 patients who had confirmed open laparotomy as an emergency, a 100% capture of all emergency laparotomy patients within the 1 year audited period. Descriptive statistics for the study cohort patient characteristics, perioperative anaesthetic management details, surgical factors and outcomes are shown in Table 1a,b. The median (25–75%) CCI score was 27 (9–45), 30-day mortality was 11.7% and 90-day mortality was 16.6%.

**Table 1:** a: Patient characteristics, perioperative details and postoperative outcomes measured, b. Perioperative administration of fluids, blood products and inotropic and vasopressor medications.

Factor of interest	N (%)	Mean ± SD or median [IQR]
*(a)*		
Gender (M: F)	47:39	
Age		63.0 ± 15.8
**Cardiac risk factors** (Lee Revised Cardiac Index)		
0 factors	26	
1 factors	22	
2 factors	18	
3 factors	16	
4 factors	4	
**Severity of pre-existing respiratory symptoms:**		
No symptoms	53	
Dyspnoea on exertion	23	
Dyspnea at rest	10	
**American Society of Anesthesiologists (ASA) Score**		
ASA 1	4	
ASA 2	22	
ASA 3	42	
ASA 4	16	
ASA 5	2	
Initial SAP (mmHg)		118 ± 29
Initial DAP (mmHg)		58 ± 17
Initial MAP (mmHg)		80 ± 19
Initial HR (beats per minute)		93 ± 18
Intraoperative SAP (mmHg)		109 ± 14
Intraoperative DAP (mmHg)		57 ± 7
Intraoperative MAP (mmHg)		75 ± 8
Intraoperative HR (beats per min)		86 ± 16
Lowest intraoperative SAP (mmHg)		72 ± 12
Lowest intraoperative MAP (mmHg)		51 ± 9
Total intraoperative urine output (mls)		497 ± 445
Postoperative Transfusion	17 [20%]	
Central venous access	69 (80%)	
Arterial line	81 (94%)	
**Emergency category**		
Emergency	38	
Urgent	48	
**Complexity of surgery**		
Major operation	37	
Complex major operation	49	
**Number of surgeries during admission**		
1	41	
2	42	
3	3	
**Peritoneal soiling**		
Grade 1	43	
Grade 2	12	
Grade 3	10	
Grade 4	21	
**Indication for surgery:**		
Intestinal obstruction	27	
Enteric perforation	39	
Gut ischaemia	14	
Incarcerated hernia	4	
Blunt trauma	2	
**Surgical procedure performed**		
Washout without resection	12	
Total colectomy	7	
Small bowel resection	22	
Hartmann’s procedure	14	
Delayed closure	2	
Other procedures	29	
**Final Surgical pathology**		
Diverticular disease	8	
Malignancy	10	
Mesenteric ischaemia	18	
Peptic ulcer disease	9	
Adhesional obstruction	15	
Incarcerated hernia	5	
Toxic megacolon	1	
NSAID induced colitis	1	
Anastomotic leak	2	
Other enteric perforation	4	
Angiodysplasia	1	
Intestinal obstruction	9	
Colocutaneous fistula	1	
Negative laparotomy	1	
Latrogenic perforation during endoscopic procedure	1	
**Stage of cancer if applicable**		
Distant metastasis	4	
Nodal metastasis	3	
Primary tumour no spread	2	
No malignancy/not applicable	77	
CT performed preoperatively	76	
CT reported preoperatively	60	
Time to theatre (h)		2.5 [2.0–4.0]
**Postoperative destination**		
ITU	25	
HDU	43	
Surgical ward	18	
Unplanned return to theatre	9 [11%]	
Unplanned critical care admission	20 [23%]	
Critical care length of stay	4 [2–10.5]	
30 day mortality	10 [12%]	
90 day mortality	14 [16%]	
Renal Replacement Therapy postoperatively	4 [5%]	
Duration of Total Parenteral Nutrition (TPN) (days)	2.8 (6.8)	
Medicine for the Elderly review postoperatively	15 [17%]	
CCI score		27 [9–45]
Overall length of stay		14 [8–31]
**Time of the day/week during which surgery was performed:**		
Daytime (0800–2000)	48	
Night-time (2000–0800)	26	
Weekend/public holiday daytime	12	

Product	No of patients receiving product n	Mean total volume administered (mls) ± SD
*(b)*		
Crystalloid	86	2041 ± 1317 mls
Colloid	14	Mean 110 + 330
RCC	23	190 ± 270
Fibrinogen	7	135 ± 45
Platelets	7	371 ± 183
Fresh frozen plasma (FFP)	10	760 ± 260
Noradrenaline	54	1.01 ± 0.90
Adrenaline	11	0.22 ± 0.17
Phenylephrine	32	310.9 ± 212
Metaraminol	39	2.3 ± 1.5
Ephedrine	7	8.6 ± 2.8

NSAID = non steroidal anti inflammatory CT = computerized tomography scan, SAP = systolic arterial pressure, DAP = diastolic arterial pressure, MAP = mean arterial pressure, HR = heart rate, ITU = intensive treatment unit, HDU = high dependency unity.

### Univariate analysis

The univariate analysis of audited variables which were potential candidate predictors of CCI are shown in Table 2. Some 25 variables correlated with CCI with the *P* < 0.05 statistical significance level, and where this occurred, the 95% confidence interval of the correlation coefficients are shown.

**Table 2:** Univariate analysis of patient characteristics, perioperative factors and postoperative parameters affecting Comprehensive Complications Index (CCI) during emergency laparotomy.

Parameter	Correlation Co-efficient (r) 95% CI where * P* < 0.05	*P* value
Age	0.13	0.22
ASA grade	0.46 (0.26 to 0.62)	0.0001
Intraoperative systolic blood pressure	− 0.3464 (− 0.5201 to − 0.1451)	0.0011
Intraoperative diastolic blood pressure	− 0.3441 (− 0.5182 to − 0.1427)	0.0012
Intraoperative mean arterial pressure	− 0.3792 (− 0.5471 to − 0.1820)	0.0003
Total intra-operative fluids	0.2776 (0.06984 to 0.4623)	0.0097
Crystalloids	− 0.1624	0.1351
Colloids	0.2871 (0.08008 to 0.4704)	0.0074
RBC transfusion (300mls)	0.4335 (0.2440 to 0.5911)	< 0.0001
Fibrinogen (50mls)	0.3846 (0.1880 0.5515)	0.0003
FFP (250mls)	0.3599 (0.1602 to 0.5313)	0.0007
Intraoperative noradrenaline in mg	0.4361 (0.2471 to 0.5932)	< 0.0001
Intraoperative adrenaline in mg	0.3253 (0.1218 to 0.5025)	0.0022
Intraoperative vasopressin in units	0.2316 (0.01942 to 0.4238)	0.033
Complexity of surgery	0.2817 (0.07430 to 0.4658)	0.0086
Number of operations within the same admission	0.3272 (0.1239 to 0.5041)	0.0021
Unplanned ICU admission	0.4243 (0.2334 to 0.5837)	< 0.0001
Mortality at 30 days	0.5598 (0.3948 to 0.6899)	< 0.0001
Mortality at 90 days	0.5703 (0.4077 to 0.6979)	< 0.0001
Dialysis	0.3412 (0.1394 to 0.5158)	0.0013
Duration of pressors post-operative	0.3666 (0.1678 to 0.5368)	0.0005
Discharge destination	0.5889 (0.4307 to 0.7119)	< 0.0001
Total length of hospital stay	0.3386 (0.1339 to 0.5155)	0.00016

### Multivariate analysis

Candidate parameters with the highest level of significance, *P* < 0.001, which were also on a prior list of biologically plausible predictors of postoperative complications, were included in a multivariate linear analysis, which is shown in Table 3. These parameters were: ASA grade, mean SABP, lowest MAP, red cell volume transfused, noradrenaline infusion total dose, escalation to an ICU bed and requirement of renal replacement therapy. In this model, the only independent predictors of CCI were ASA status and unplanned escalation of postoperative management to ICU.

**Table 3:** Multivariable linear regression analysis of factors associated with Comprehensive Complications Index (CCI).

Parameter	Univariate Analysis	Multivariate Linear analysis: Estimate (95% CI)	*P* value
ASA Grade	0.0001	14.1 [4.3–23.9]	0.005
Average SAP	0.001	− 0.9 [− 1.5 to 2.2]	0.89
Lowest MAP	0.003	− 0.04 [− 0.6 to 0.5]	0.80
Red cell volume	0.0001	− 0.65 [− 5.3 to 3.9]	0.77
Noradrenaline infusion dose	0.001	0.9 [− 7.5to 9.3]	0.83
Escalation to ITU	0.0001	8.0 [1.6–33.2]	0.032
Needing renal replacement therapy	0.001	0.13 [− 0.14–0.37]	0.38

30 day mortality after surgery was examined as a secondary outcome. Again, from among the variables most strongly associated with 30-day mortality from the univariate analysis, those factors considered biologically plausible were included in a multivariate logistic regression model. These are shown in Table 4, displayed as Odds Ratio (OR) with 95% CI. Again, only age, lowest intraoperative MAP, transfusion volume of red cells and CCI score were independently associated with 30-day mortality.

**Table 4:** Multivariable logistic regression factors associated with 30-day mortality.

Parameter	Odds Ratio (95% CI)	*P* value
Age	1.00 [0.93–1.08]	0.67
ASA status	3.06 [1.00–14.1]	0.05
Lowest MAP	1.05 [1.01–1.13]	0.04
Red cell transfusion volume	1.91 [1.20–4.50]	0.02
Noradrenaline infusion total dose	1.14 [0.45–1.70]	0.49
Urgency of surgery	2.40 [0.34–20.1]	0.71
Escalation to ITU	1.7 [0.23–12.2]	0.76
Needing renal replacement therapy	1.2 [0.4–1.9]	0.44

In addition to analysis of the correlation between perioperative parameters and post-operative morbidity and mortality we sought to compare our data previously published through the NELA in the UK. The findings of this comparison are presented in Table 5.

**Table 5:** Comparison of current study data versus NELA data

Parameter	Current study	First NELA report	Fifth NELA report
Number of cases	86	39,908	24,328
Mean age	64	68	67 (median)
30 day mortality	12%	11.3%	9.6%
90 day mortality	16%	15.5%	13%
Proportion of patients with ASA > III	70%	56%	55%
Total length of stay	14	19.2	16
Number admitted to critical care	77%	60%	77.5%
Patients receiving pre-operative CT	87%	n/a	88.5%
Patients reviewed by medicine for the elderly team post operatively	32.4%	10%	22.5%

## Discussion

The UK National Emergency Laparotomy Audit (NELA) began in 2013 following the AAGBI consensus statement on emergency general surgery^10^. This report identified a number of concerns with regard to the provision of emergency surgical care particularly in terms of systems and organisational structure. It was recognised that, in contrast to elective services, emergency general surgery was under-resourced and under-researched, despite the higher patient risk profile^10^. The first NELA was conducted to identify specific areas requiring quality improvement in the provision of emergency surgical care through laparotomy. The cycle has been repeated on an annual basis and has shown a trend towards greater adherence to defined standards with each cycle^4,11,12^.

In our cohort, age, length of stay and rates of admission to critical care were all in line with NELA rates which have stayed relatively static since the beginning of the audit^4,11,12^. However, our study showed a greater proportion of high-risk patients, as defined by higher ASA score of 3 or above (74% compared to 56%^11^ and 55%^4^). This may be due to the status of our hospital as a tertiary referral centre compared to the large number of model 3 hospitals included in NELA. Despite this, our mortality rate compares favorably to the initial NELA rate (11.6% vs. 14.9%)^11^ but trails the most recent NELA rate of 9.6%^4^. Availability of abdominal CT was specifically targeted by the 2007 ASGBI report as an area requiring improvement. Our study found 87% of patients undergoing CT prior to theatre with 69% of CT reports being issued by a consultant radiologist prior to theatre. This again compares favourably with the most recent NELA data of 88% and 62% respectively^4^.

Both the Association of Anaesthetists and NELA reports emphasise the importance of the presence of consultant staff in theatre during an emergency laparotomy. The most recent NELA report showed the vast majority of emergency laparotomies had a consultant surgeon (92%) and anesthesiologist (88%) present in theatre^4^. The presence of consultants in theatre was not reliably documented in our electronic records and so we were unable to gather data for comparison.

Another area targeted by NELA is input from a Medicine for the Elderly service perioperatively for patients > 70 years. Elderly patients are more likely to have a greater quantity and severity of co-morbidities, reduced physiological reserve to withstand surgery and are more likely to have evidence of frailty compared with younger populations^13^. The time sensitive nature of emergency surgery restricts optimisation of these co-morbidities as would routinely be done in the elective setting for these patients. The first NELA report found only 10% of patients over the age of 70 were assessed by a medicine for the elderly specialist, which improved to 22.5% subsequently^4,11,12^. Our rate of 32% compares favourably to this. This population group will continue to be of increasing significance as our population continues to age^14^.

Previously published studies looking at perioperative factors have focused primarily on mortality as an outcome^5,15,16^. However, it has been well established that emergency surgery has a high risk of postoperative complications other than death, ranging from relatively minor complications such as ileus to severe complications, such as wound infection or stroke^17,18^. To address this, we chose Comprehensive Complication Index (CCI) as an outcome measurement.

While ASA grade was predictably associated with increased CCI, our data did not show age to be correlated with it. This may have been a chance finding related to our relatively small patient cohort. Hypotension was also negatively correlated with CCI. This observation is consistent with current observational studies which have associated intraoperative hypotension with myocardial injury after non cardiac surgery and mortality^6,7^. Hypotension may indicate organ hypoperfusion leading to postoperative organ impairment and clinical morbidity.

The volume of intra-operative fluids, colloids, RCC, fibrinogen, fresh frozen plasma, noradrenaline, adrenaline and vasopressin were all positively correlated with CCI, which is likely a marker of severe surgical physiological disturbance occurring intraoperatively. As a result, these factors interact with each other. However, the correlation between ASA grade, lowest MAP, units of RCC transfused and increased CCI persisted following multivariate analysis. This correlation could be considered when deciding the appropriate level of care for patients undergoing emergency laparotomy post operatively as it has been previously shown that improved nurse to patient ratio (as seen in ITU compared with standard surgical ward) is associated with better detection of post-operative complications and reduced mortality^19^.

While complexity of surgery and number of operations on admission positively correlated with CCI on univariate analysis, this did not persist on multivariate analysis, suggesting this was due to confounding factors. Interestingly, the indication for surgery, procedure performed and ultimate surgical pathology did not show a correlation with CCI. This is in contrast to what has been published previously with a number of studies showing markedly worse outcomes for patients undergoing emergency laparotomy for mesenteric ischaemia in particular^4,5,11,20^.

In addition to examining physiological factors associated with increased CCI, we also examined systems factors that may influence post-operative morbidity. Of the organisational factors measured, post-operative destination was the only one bearing a relationship to CCI, with ITU patients incurring a higher CCI than those fit for ward management. This finding has previously been shown with regard to mortality rates^15^. While initially there was a correlation between day of the week and CCI this did not persist with multivariate analysis.

The provision of acute surgical care is currently under considerable debate both in Ireland and abroad as elective services are becoming more centralised. A number of studies have shown specialist surgeons to have better outcomes than non-specialists when it comes to both elective and emergency surgery^21–24^. The volume of emergency surgeries performed by a surgeon is also shown to correlate with improved outcomes^25,26^. Our study highlights the importance of high quality critical care facilities for any hospital participating in general emergency surgery.

A strength of our study is the use of electronic records for all theatre and critical care records in addition to discharge summaries at our institution. However, non-critical care clinical notes are not part of the electronic record. Improved access to these records would enable more accurate data collection, particularly regarding administration of antibiotics, consultant review and the presence of more minor post-operative complications, which may not be included in the discharge summary. Although our data is from a single centre, our cohort of patients was diverse. While this may impact our increased ASA score compared to the NELA data (which includes a significant number of level 3 hospitals), it is advantageous in that patients from all socioeconomic backgrounds with varying levels of complexity are represented in our study. This is relevant as a hospital providing an emergency general surgery service must be equipped to deal with this mixed patient cohort. Our study is limited by its retrospective nature and sample size being relatively small.

In conclusion, this single-centre retrospective analysis shows an association between ASA grade, volume of red cells transfused and lowest MAP with increased morbidity in the form of increased CCI. These parameters should be considered along with other predictive factors when making decisions for post-operative management. From our study we can conclude that the provision of emergency laparotomy in this tertiary referral, university teaching hospital compares favourably with the NELA targets with regard to mortality rate, critical care admission and preoperative imaging. However, improvements can be made in the area of postoperative medicine for the elderly review. This highlights the need for a prospectively maintained database, at national level, to ensure we are meeting international standards and to assist in the planning of acute surgical care.
